# In and Outpatients Bacteria Antibiotic Resistances in Positive Urine Cultures from a Tertiary Care Hospital in the Western Part of Romania—A Cross-Sectional Study

**DOI:** 10.3390/diseases13030074

**Published:** 2025-03-01

**Authors:** Nicu Olariu, Monica Licker, Lazar Chisavu, Flavia Chisavu, Adalbert Schiller, Luciana Marc, Oana Albai, Andrei Paunescu, Vlad Tucicovschi, Adelina Mihaescu, Adrian Apostol

**Affiliations:** 1 Centre for Molecular Research in Nephrology and Vascular Disease, “Victor Babes” University of Medicine and Pharmacy-Faculty of Medicine, Eftimie Murgu Square No. 2, 300041 Timisoara, Romania; nicu.olariu@umft.ro (N.O.); farkas.flavia@umft.ro (F.C.); schiller.adalbert@umft.ro (A.S.); marc.luciana@umft.ro (L.M.); albai.oana@umft.ro (O.A.); mihaescu.adelina@umft.ro (A.M.); adrian.apostol@umft.ro (A.A.); 2 Dialysis Compartment, “Pius Brinzeu” Emergency Clinical County University Hospital, 300723 Timisoara, Romania; 3 Microbiology Department, Multidisciplinary Research Center of Antimicrobial Resistance, “Victor Babes” University of Medicine and Pharmacy, Eftimie Murgu Square No. 2, 300041 Timisoara, Romania; licker.monica@umft.ro; 4 Microbiology Laboratory, “Pius Brinzeu” Emergency Clinical County University Hospital, 300723 Timisoara, Romania; 5 Nephrology Discipline, “Victor Babes” University of Medicine and Pharmacy, Eftimie Murgu Square No. 2, 300041 Timisoara, Romania; 6 “Louis Turcanu” Emergency County Hospital for Children, 300011 Timisoara, Romania; 7 Department of Second Internal Medicine Diabetes, Nutrition, Metabolic Diseases, and Systemic Rheumatology, “Victor Babes” University of Medicine and Pharmacy, 300041 Timisoara, Romania; 8 Department of Diabetes, Nutrition and Metabolic Diseases Clinic, “Pius Brînzeu” Emergency Clinical County University Hospital, 300723 Timisoara, Romania; 9 Department of Urology, “Pius Brînzeu” Emergency Clinical County University Hospital, 300723 Timisoara, Romania; ai.paunescu@gmail.com; 10 PhD Department,“Victor Babes” University of Medicine and Pharmacy, Eftimie Murgu Square No. 2, 300041 Timisoara, Romania; vlad.tucicovschi@umft.ro; 11 Department of Nephrology, “Pius Brînzeu” Emergency Clinical County University Hospital, 300723 Timisoara, Romania; 12 Department of Cardiology, “Pius Brînzeu” Emergency Clinical County University Hospital, 300723 Timisoara, Romania; 13 Cardiology Discipline, “Victor Babes” University of Medicine and Pharmacy, Eftimie Murgu Square No. 2, 300041 Timisoara, Romania

**Keywords:** in and outpatients, antibiotic resistance, multidrug-resistant bacteria, positive urine culture

## Abstract

Background/Objectives: Urinary tract infections (UTI) represent a global problem with implications for mortality and morbidity. Published data present different bacterial incidences and different antibiotic resistance. The objective of our study is to evaluate the bacteria distribution in positive urine cultures in a mixed adult population and evaluate the differences in antibiotic resistance in in- and outpatients. Methods: We analyzed 1186 positive urine cultures in 2021 from the Emergency County Hospital “Pius Brinzeu” from Timisoara, Romania. We evaluated the bacteria distribution and antibiotic resistance stratified by in and outpatients from a mixed adult population. Results: The median age was 67, with 65.7% females and 28.5% were outpatients. In inpatients, the most commonly identified bacteria was *E. coli*, followed by *Enterococcus* spp., and *Klebsiella* spp., while in outpatients, *E. coli*, *Enterococcus* spp., and *Klebsiella* spp. were the leading ones. Overall, *E. coli* presented the highest resistance rate to ampicillin, *Enterococcus* spp. to ciprofloxacin, *Klebsiella* spp. to cephalosporins, and *Proteus* spp. to trimethoprim/sulfamethoxazole. Inpatients presented higher resistance rates for *E. coli* to ceftazidime, cefuroxime, gentamycin, ciprofloxacin, and trimethoprim/sulfamethoxazole, *Klebsiella* spp. to most cephalosporin, gentamycin and levofloxacin, *Proteus* spp. to gentamycin and *Enterococcus* spp. to gentamycin and quinolones when compared to outpatients. The highest incidence of extensively drug-resistant (XDR) bacteria was among *Acinetobacter baumanii*, followed by *Pseudomonas* spp., and *Serratia* spp. Conclusions: susceptibility. Bacteria identified in inpatients’ positive urine cultures present higher resistance rates to several antibiotics. Our study could be a foundation for a local or even national guideline for the antibiotic treatment of urinary tract infections.

## 1. Introduction

Multidrug-resistant bacteria (MDR) is one of the major public health problems of the 21st century. The most comprehensive and latest published data regarding worldwide antimicrobial resistance reported more than 5 million deaths in 2019 attributed to resistance, and more than 1.25 million people had a death directly linked to multidrug-resistant bacteria (MDR) [1]. Many other studies proved that MDR bacteria increase mortality and morbidity [2,3,4,5,6]. On the other hand, the rise of antibiotic resistance is associated with increased economic burn, hospitalization, and higher disability-adjusted life-years [1,2]. In the face of all these implications, one should constantly assess and evaluate the regional antibiotic resistance patterns to identify possible solutions to reduce MDR mortality and economic impact [1].

Real data regarding antimicrobial resistance rates of positive urine cultures from Romania are scarce and the focus was mainly on female patients [7,8,9,10,11,12]. *Escherichia coli* was the leading bacteria to generate a urinary tract infection followed by *Klebsiella* and *Proteus*. These bacteria remained the most commonly identified pathogens in patients with diabetes mellitus from the Western part of Romania who developed UTI [13,14].

Data from Romania report different antimicrobial resistance rates in patients with UTI [9,10,11,12,13]. For instance, *E. coli* resistance to quinolones varies from 30 to 72%, and penicillin’s between 14 and 66% [7,9]. For *E. coli*, Petca et al. reported the highest resistance rate to ciprofloxacin (30%) and only 14% to amoxicillin–clavulanic acid in females [7], much lower than Chibelean et al., who reported a 72% resistance to quinolones and 66% to penicillin-amines in males [9]. In an analysis of six East European countries, *E. coli* presented the highest resistance rate to ampicillin (39.6%) and trimethoprim (23.8%), and around 15% to ciprofloxacin [15]. These differences in resistances are consistent with other bacteria, like *Klebsiella*, *Enterobacter*, or *Pseudomonas* [7,8,9,10,11]. These heterogeneous results are the consequence of the empirical antibiotic therapy used in treating UTIs. Even though there are European guidelines recommending a “safe” use of an antibiotic if the resistance is lower than 20%, real-life data show that it is impossible for a continental guideline to be adequate.

In the face of such scattered data regarding antibiotic resistance, and the lack of national guidelines for antibiotic treatment of UTI, there is an imperative need to assess the antimicrobial resistance rates depending on the geographic area before the physician can prescribe an empirical treatment for UTI. Otherwise, the ascending trend of improper use of certain antibiotics will influence antimicrobial resistance rates, especially in patients with multiple UTIs. Nevertheless, every region could have a different approach regarding the type of antibiotics used in treating infections, if there is enough information regarding the local antimicrobial resistance.

Studies from the western part of Romania on UTI bacteria are scarce and they evaluate specific populations like children or patients with diabetes mellitus [13,14,16].

There is a known fact that most of the bacteria responsible for UTIs are the Gram-negative ones [7,8,9,10,11,12]. Nevertheless, the incidence of UTIs generated by Gram-positive bacteria can be up to 30% [9]. On the other hand, some bacteria strains are more common in UTI, as presented earlier.

To fill this gap, we performed a retrospective cross-sectional analysis in the largest hospital in the western part of Romania on positive urine cultures identified during 2021 in a mixed adult population. We aimed to evaluate the bacterial resistance to the most used antibiotics, to identify differences in resistance, and to determine the incidence of MDR bacteria. In our analysis, we included the positive urine cultures obtained from inpatients and outpatients as a comparative focus. Due to different antibiotic susceptibility, we classified bacteria according to Gram classification in Gram-positive and Gram-negative. In addition, we explored the differences regarding antibiotic resistance for most common bacteria.

## 2. Materials and Methods

### 2.1. Data Collection

We performed a cross-sectional retrospective analysis of all the positive urine cultures from the Emergency County Hospital “Pius Brinzeu” from Timisoara, Romania identified in the year 2021. The “Pius Brinzeu” Emergency County Hospital from Timisoara Ethics Committee approved the study (466/17 May 2024) and it was performed in accordance with the Ethics Code of the World Medical Association. The study followed the recommendations of the Declaration of Helsinki. The inpatients signed an informed consent at the admission to the hospital. The outpatients signed an informed consent at the time of handing over the urine sample. We used the electronic data system to extract all the positive urine cultures. We recorded the gender, age, and the identified bacteria. In addition, we extracted the susceptibilities and resistances to several antibiotics. In the analysis, we included only the patients older than 18 years. We analyzed only the first positive urine culture (if a patient presented multiple positive urine cultures) and included only single germ cultures. Out of 41,027 evaluations during the year 2021, we retrieved 1186 positive urine cultures.

### 2.2. Sample Collection

The samples were collected over a 1-year, comprising all of the positive urine cultures from patients used to confirm the clinical suspicion of infection. All the details regarding the sample collection are presented in the Supplemental Materials.

### 2.3. Bacterial Identification and Antibiotic Testing

Collection of specimens was performed according to our hospital’s Specimen Collection Manual (https://www.hosptm.ro/sectiile-spitalului/laborator-clinic-de-analize-medicale/, accessed on 17 December 2024). All the details regarding bacterial identification and antibiotic testing are explained in the Supplemental Materials. The antibiotics tested are listed in Supplementary Tables S1 and S2. AST interpretation was performed according to the Clinical Laboratory and Standards Institute (CLSI) [17,18]. Multidrug-resistant (MDR) bacteria have been considered resistant to at least one antibiotic from three or more classes active for a given species [19]. Extensively drug-resistant bacteria (XDR) were considered resistant to at least one antibiotic from all tested classes but maintained susceptibility in agents from maximum of 2 antibiotic categories [19].

Classification into resistance phenotypes was performed according to CLSI criteria [14]. We used the following reference strains: *Escherichia coli* ATCC 25922, *Klebsiella pneumoniae* ATCC1705, *Pseudomonas aeruginosa* ATCC 27853, and *Staphylococcus aureus* ATCC 25923.

### 2.4. Statistical Analysis

The data are presented as numbers and percentages for categorical variables. The age distribution was non-Gaussian (evaluation performed using the Shapiro–Wilk test) and is presented as the median and interquartile range (IQR). The chi-square test was used for categorical variables to evaluate the distribution of certain antibiotic resistance between in- and outpatients. The test was unadjusted. A *p*-value less than 0.05 was considered statistically significant. We used the Mann–Whitney test to evaluate the age-stratified by in- and outpatient status. MedCalc^®^ Statistical Software version 22.021 (MedCalc Software Ltd., Ostend, Belgium; https://www.medcalc.org, accessed on 17 December 2024) was used to perform the analysis.

## 3. Results

**Figure 1:**
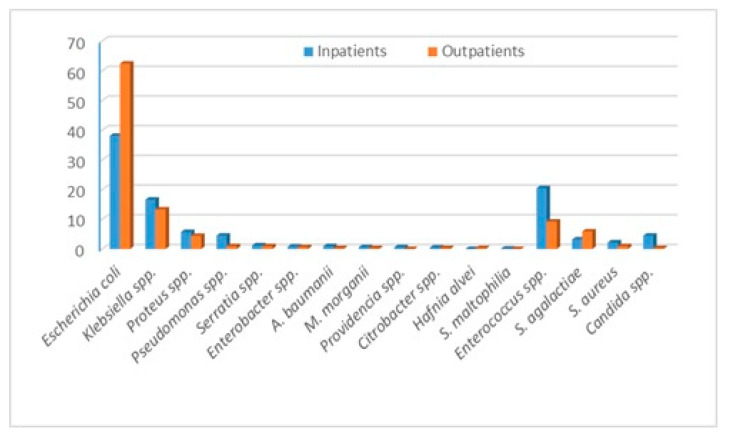
Bacteria distribution stratified by in and outpatients.

The bacteria that we identified and evaluated are *Escherichia coli* (*E. coli*), *Klebsiella* spp., *Proteus* spp., *Pseudomonas* spp., *Staphylococcus aureus*, *Enteroccocus* spp., *Enterobacter* spp., *Acinetobacter baumanii*, *Providencia* spp., *Citrobacter* spp., *Morganella morganii*, *Hafnia alvei*, *Stenotrophomonas maltophilia* and *Streptococcus agalactiae*. The outcomes of our study are the bacteria distribution and the incidence of certain antibiotic resistance and susceptibilities, overall and stratified by in and outpatients. In addition, we evaluated the multidrug resistance of the most commonly identified bacteria. During the year 2021, in the “Pius Brinzeu” Emergency County Hospital from Timisoara, there were 41,027 admissions. There were 1186 positive urine cultures. The bacteria distribution is presented in Table 1. *Escherichia coli*, followed by *Enterococcus* spp., *Klebsiella* spp., and *Proteus* spp., were the most common bacteria in the entire cohort and in in-patients. The outpatients presented the highest incidence of *E. coli*, followed by *Klebsiella* spp., *Enterococcus* spp., and *Streptococcus agalactiae*. *Candida* spp. presented a much higher incidence among in-patients Figure 1. Inpatients presented more comorbidities, and some of them developed UTIs during admission. Thus, they were at higher risk of infection with “hospital bacteria”.

**Figure 2:**
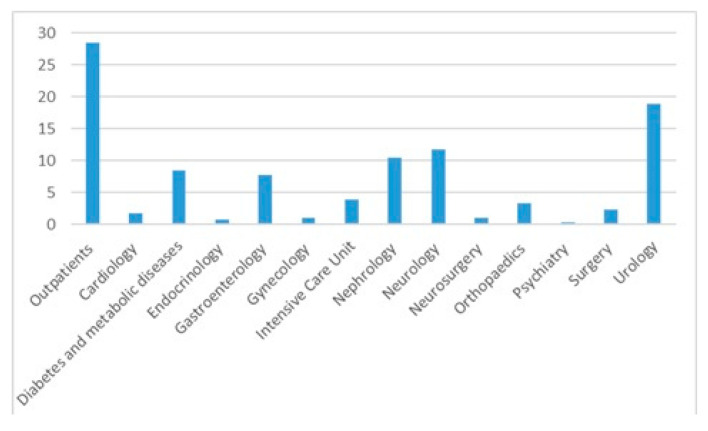
Urine cultures distribution per department.

The median age was 67 years (IQR = 56–75), with 779 females (65.7%) females. The in-patients were older compared with the outpatients (median age and IQR 68 [59–76] vs. 65 [48–72], *p* < 0.0001). Most positive cultures were from outpatients (28.5%) followed by cultures from the urology (18.8%) and neurology departments (11.7%)—Figure 2 and Table 2. Out of the entire positive cultures, 145 (12.2%) patients had diabetes mellitus.

### 3.1. Overall Resistance

#### 3.1.1. Gram-Negative Bacteria

To evaluate bacteria’s susceptibilities to the tested antibiotics, we performed a stratified analysis by bacteria. To ensure non-biased results, we included only the strains tested for a specific antibiotic. The presented percentages represent the resistance rates, and the difference to 100 is the susceptibility—Table 3.

*Escherichia coli* presented the highest antimicrobial resistance rates to ampicillin—76.9%, amoxicillin–clavulanic acid—42.3% followed by trimethoprim–sulfamethoxazole—35.6%, quinolones (28.2% to ciprofloxacin and 27.7% to levofloxacin) and cephalosporin (20.2% to cefuroxime, 19.3% to ceftriaxone and 16.9% to cefepime). On the other hand, the resistance rate to aminoglycosides was low (8.4% to gentamycin and 1.7% to amikacin).

*Klebsiella* spp. presented a heterogeneous pattern of resistance to commonly used antibiotics: around 40% resistance rates to cephalosporins, more than 30% to quinolones, and 13.7% to carbapenems.

*Proteus* spp. presented the highest resistance rate to trimethoprim–sulfamethoxazole (45.8%), 2nd and 3rd generation cephalosporins (16% to cefuroxime and 22.2% to ceftazidime), 25–30% to quinolones and 30.4% to gentamycin. One should mention that only two strains presented resistance to imipenem and 7.7% to 4th generation cephalosporin.

*Pseudomonas* spp. presented high resistance rates to quinolones (50%), cephalosporin (35–40%), 41.7% to gentamycin and 32.4% to carbapenems.

#### 3.1.2. Gram-Positive Bacteria

*Enterococcus* spp. presented extremely high resistance rates to quinolones (60–70%), 36.7% to gentamycin, and 16.1% to ampicillin. Only two strains were resistant to vancomycin and none to linezolid —Table 4.

*S. aureus* presented high resistance rates to erythromycin (62.5%), quinolones (38.9% to ciprofloxacin), and 26.3% to gentamycin. Resistance to linezolid was 0%—Table 4.

*S. agalactiae* presented high resistance to clindamycin (87.5%), erythromycin (43.7%), and levofloxacin (44.2%)—Table 4.

### 3.2. In- and Outpatient Resistance

To have a clear image of resistance, we evaluated the resistance distribution of the most common bacteria stratified by in and outpatients. We analyzed *E. coli, Klebsiella* spp., *Proteus* spp., and *Enterococcus* spp.

#### 3.2.1. Gram-Negative Bacteria

*E. coli* presented higher resistance rate in in-patients compared to outpatients to ampicillin (100% vs. 65.8%), ceftazidime (19.1% vs. 9.3%), cefuroxime (22.6% vs. 11.5%), gentamycin (10.8% vs. 4.7%), ciprofloxacin (32.6% vs. 22.2%) and trimethoprim/sulfamethoxazole (39.8% vs. 29.2%). *Klebsiella* spp. presented higher resistance rate to most cephalosporin (43.9% vs. 13.6% for ceftazidime and 42.3% vs. 12% for cefuroxime), gentamycin (23.4% vs. 2.4%) and levofloxacin (35.4% vs. 10.5%) in inpatients compared with outpatients. *Proteus* spp. presented a higher resistance rate in inpatients only to gentamycin (38.1% vs. 7.1%)—Table 5.

#### 3.2.2. Gram-Positive Bacteria

*Enterococcus* spp. presented higher resistance rates to gentamycin (41.7% vs. 9.7%) and all quinolones (72.1% vs. 42.9% for ciprofloxacin and 65.9% vs. 29.2% for levofloxacin) in inpatients compared to outpatients—Table 6.

### 3.3. Resistance Patterns

*Acinetobacter baumanii* presented the highest resistance rates, and almost half of the evaluated strains were extensively drug-resistant (XDR). *Pseudomonas* spp. presented a 26.8% incidence of XDR, and all evaluated strains produce beta-lactamase and *Serratia* spp. 15.3%. In addition, it presented high fluoroquinolone and aminoglycosides resistance. *Acinetobacter baumanii* was the most common ESBL-producing bacteria and carbapenem-resistant, followed by *Klebsiella* spp. and *Pseudomonas* spp. *Proteus* spp. was the leading pathogen to sulfonamide resistance. Almost half of *Klebsiella* spp. strains produce ESBL, with a third being MDR, but only 4.8% XDR. MRSA incidence was 35.3% (6/17), and two-thirds of *S. aureus* strains were MDR—Table 7.

## 4. Discussion

Our study is the first one to evaluate bacteria and the antibiotic resistance rate identified in a mixed adult population stratified by in and outpatients from the Western part of Romania. The most commonly identified bacteria is *E. coli*, followed by *Enterococcus* spp., *Klebsiella* spp., and *Proteus* spp. Outpatients presented a slightly different pattern, with *E. coli* representing almost two-thirds of identified bacteria, followed by *Klebsiella* spp. and *Enterococcus* spp.

*E. coli* presented the highest resistance rate to amoxicillin–clavulanic acid, *Enterococcus* spp. to quinolones, *Klebsiella* spp. to cephalosporins, and *Proteus* spp. to trimethoprim–sulfamethoxazole. Inpatients presented the highest resistance rates for *E. coli* to ampicillin, ceftazidime, cefuroxime, gentamycin, ciprofloxacin, and trimethoprim/sulfamethoxazole, *Klebsiella* spp. to most cephalosporins, gentamycin and levofloxacin, *Proteus* spp. to gentamycin and *Enterococcus* spp. to gentamycin and all quinolones. The highest incidence of XDR bacteria was among *Acinetobacter baumanii*, followed by *Pseudomonas* sppand *Serratia* spp. Almost half of *Klebsiella* spp. strains produce ESBL but only 4.8% XDR. MRSA incidence was 35.3%.

The female gender is associated with a higher risk of urinary tract infection (UTI) especially before the age of 50 years old [20]. In the elderly, it seems that both females and males have the same risk of UTI, especially due to the urological-associated pathology in males [21,22]. Even with a relatively advanced age (67), our cohort has a slight predominance of females (65.7%).

On the other hand, we identified some differences regarding the bacteria distribution in in- and outpatients. *E. coli* presented a higher percentage among outpatients (62.4% vs. 38.1%). In inpatients, *Enterococcus* spp. and *Klebsiella* spp. were the next most common bacteria, while in outpatients, this order was reversed. The inpatients present several risk factors that increase the risk of nosocomial infection, like older age, multiple comorbidities, urine catheters, multiple antibiotics administration, and invasive procedures. For instance, the fact that some of the inpatients presented urine catheters explains the higher incidence of *Pseudomonas* spp. in this group. These risk factors increase the risk of infection with MDR agents, and thus, one should expect a much higher incidence of these bacteria in the inpatients. All of these factors should influence the decision of empirical antibiotherapy. For outpatients, the doctors could choose from a larger number of antibiotics to treat a UTI, but for the inpatients, the treatment possibilities are lower due to a higher incidence of MDR bacteria.

Sorescu et al. recently evaluated bacteria distribution in urinary tract infections from patients with diabetes from the same hospital as ours [14]. The trend was similar, with *E. coli* being the leading pathogen, followed by *Klebsiella* spp. and *Enteroccocus* spp. [14].

Several factors are involved in bacterial resistance development [23]. In addition to the increased use of antibiotics in both people and animals, there is a link between resistance to different antibiotics and the most commonly prescribed antibacterial agents within different regions [23].

Previously published data from Romania reported different susceptibilities in both males and females. For *E. coli*, Petca et al. reported the highest resistance rate to ciprofloxacin (30%) and only 14% to amoxicillin–clavulanic acid in females [7], much lower than Chibelean et al., who reported a 72% resistance to quinolones and 66% to penicillin amines in males [9]. In an analysis of six East-European countries, *E. coli* presented the highest resistance rate to ampicillin (39.6%) and trimethoprim (23.8%), and around 15% to ciprofloxacin [15].

We report a higher resistance rate to levofloxacin for *Enterococcus* spp. compared with current Romanian data—32% resistance to levofloxacin in females [7] and 25% to levofloxacin in males [9]. Petca et al. reported a resistance rate of 28% to amoxicillin–clavulanic acid and 15% to levofloxacin for *Klebsiella* spp. in females [7], while Chibelean et al. 59% to amoxicillin–clavulanic acid, 44% to levofloxacin and 38% to ceftazidime in males [9], results different from ours. On the other hand, all of the aforementioned studies reported low resistances to carbapenems, similar to our results [7,9]. Regarding *Proteus* spp. and *S. aureus*, our results are in concordance with the current literature [7,9]. In Romania, *Pseudomonas* spp. has been associated with different resistance rates: from 31 to 44% for levofloxacin, 14 to 32% for amikacin, and 24 to 26% for ceftazidime [7,9].

As we can see, there are differences and similarities regarding bacteria resistance to several antibiotics, within the same country. One of the major factors that generates these results is the empirical antibiotic prescription for treating UTIs. On the other hand, the studied population differs, with some studies evaluating only females and others only males [7,9].

The antibiotic resistance analysis for in and outpatients represents the highlight of our study. We identified that there are some differences regarding resistance to most commonly used antibiotics. First, the inpatients were older compared to outpatients and most likely presented higher comorbidity rates. An indirect proof of this is represented by the department distribution of identified positive urine cultures. Patients admitted to the urology department have urological problems and are prone to invasive procedures on the urinary tract. Neurological patients present a high rate of immobilization, are prone to dehydration, and have a higher incidence of urine catheters. Nevertheless, more than 10% of our patients present diabetes mellitus, a known risk factor for UTIs.

Secondly, inpatients present a higher risk of hospital-acquired infections (HAI) due to invasive procedures, prolonged hospitalization, and multiple antibiotic courses. HAI infections present a higher incidence of MDR bacteria; thus, one should expect higher resistance rates [1,2,3,4,5]. We are aware that we did not evaluate the incidence of HAI in our cohort.

Worldwide, the trend of antimicrobial resistance of different uropathogens is rising. The downside seems to be a reduced susceptibility to the most common antibiotics for both community—and hospital-acquired UTIs [1,24,25].

Antibiotic resistance represents a major world health problem with high morbidity and mortality [1,26]. The reported mortality associated with MDR bacteria is rising with an estimated mortality of 5 million deaths in 2019 and it is presumed to double by 2030 [1,2]. On the other hand, the estimated costs of treating MDR bacteria represent USD 12.61 billion in 2024, with an increase of 6.5% per year, up to USD 16.22 billion in 2028 [27]. The published data regarding MDR bacteria and even the resistance to several antibiotics are not uniform, as most of the studies were conducted in developed countries [1,2,3,4,5]. Nevertheless, due to the regional pattern of antibiotic resistance, one could not properly estimate bacteria resistance to a specific antibiotic not even in the same country. However, besides the economic burden and the high mortality rates, the importance of reporting antimicrobial resistance and MDR strains is highlighted by the necessity of broad-spectrum therapeutics [28,29].

As expected, the hospital bacteria presented the highest resistance rates. *Acinetobacter baumanii* presented the highest incidences in most of the resistance patterns. It was the most common bacteria with an XDR pattern, followed by *Pseudomonas* spp. and *Serratia* spp. More than 20% of *E. coli* strains were MDR and a third of *Klebsiella* spp. A recent analysis of Petca regarding the MDR uropathogens in Romania showed an incidence of 4.5% of MDR bacteria [8]. Nevertheless, in Petca’s study, *E. coli* and *Klebsiella* spp. represented the most common MDR bacteria, while in ours were *Klebsiella* spp., *Acinetobacter baumanii*, and *Serratia* spp.

Our study presents some limitations. The cross-sectional and retrospective type, without data regarding hospital stay, mortality, and other comorbidities, could limit the impact of our results. Unfortunately, molecular testing to identify antibiotic resistance genes or virulence genes was not performed. In future studies, we plan to evaluate isolates in this perspective as well. On the other hand, a relatively high number of evaluated positive urine cultures doubled by the evaluation of the MDR incidence and stratification on in and outpatients represents the strongest points. Our study is the first one from the western part of Romania, which analyzed the positive urine culture from a tertiary care hospital.

## 5. Conclusions

In conclusion, bacteria distribution in positive urine cultures seems to follow a similar pattern regarding the region of recorded data, with some differences between in and outpatients. Because antibiotic resistance is different between countries and even in the same country, our study could be a foundation for a possible future protocol used in our hospital to initiate appropriate empiric treatment in patients with UTIs. In order to do so, one should implement programs of targeted education for physicians or local stewardship guidelines. For instance, in our region, local resistance trends for each ward/hospital should be known, and empirical therapy should be based on broad-spectrum antibiotics effective against the most common uropathogens. Therefore, we should reassess the use of quinolones, extended-spectrum cephalosporins, or trimethoprim/sulfamethoxazole as a first-line agent, especially in patients with risk factors for MDR bacterial infection.

## Figures and Tables

**Table 1:** Bacteria distribution stratified by in and outpatients.

Germs	Total N = 1186	Inpatients N = 848	Outpatients N = 338	*p* Value *
*Escherichia coli*	534 (45%)	323 (38.1%)	211 (62.4%)	<0.0001
*Klebsiella* spp.	186 (15.7%)	141 (16.6%)	45 (13.3%)	0.1568
*Proteus* spp.	63 (5.3%)	48 (5.7%)	15 (4.4%)	0.397
*Pseudomonas* spp.	41 (3.5%)	38 (4.5%)	3 (0.9%)	0.0022
*Serratia* spp.	13 (1.1%)	10 (1.2%)	3 (0.9%)	0.6633
*Enterobacter* spp.	9 (0.8%)	7 (0.8%)	2 (0.6%)	0.6755
*Acinetobacter baumanii*	9 (0.8%)	8 (0.9%)	1 (0.3%)	0.2463
*Morganella morganii*	6 (0.5%)	5 (0.6%)	1 (0.3%)	0.5199
*Providencia* spp.	5 (0.4%)	5 (0.6%)	0 (0%)	0.1573
*Citrobacter* spp.	5 (0.4%)	4 (0.5%)	1 (0.3%)	0.6732
*Hafnia alvei*	1 (0.08%)	0 (0%)	1 (0.3%)	0.1132
*Stenotrophomonas maltophilia*	1 (0.08%)	1 (0.1%)	0 (0%)	0.5278
*Enterococcus* spp.	205 (17.3%)	174 (20.5%)	31 (9.2%)	<0.0001
*Streptococcus agalactiae*	47 (4%)	27 (3.2%)	20 (5.9%)	0.0295
*Staphylococcus aureus*	22 (1.9%)	19 (2.2%)	3 (0.9%)	0.1192
*Candida* spp.	39 (3.3%)	38 (4.5%)	1 (0.3%)	0.0003

Legend: * = Chi-square test, N = number.

**Table 2:** Urine cultures distribution per department.

Department	Number and %
Outpatients	28.5% (338)
Cardiology	1.7% (20)
Diabetes and metabolic diseases	8.5% (101)
Endocrinology	0.75% (9)
Gastroenterology	7.7% (91)
Gynecology	1% (12)
Intensive Care Unit	3.9% (47)
Nephrology	10.4% (123)
Neurology	11.7% (139)
Neurosurgery	1% (12)
Orthopedics	3.3% (39)
Psychiatry	0.25% (3)
Surgery	2.3% (28)
Urology	18.8% (223)

**Table 3:** Gram-negative-resistance percentages.

Bacteria	AMC	AMP	PIP	PTZ	TIC	CFZ	CFP	CFR	CFX	IMI	MER	AMI	GEN	CIP	LEV	NOR	COL	TRS
Overall	43.2% (16/37)	76.9% (183/238)	31.1% (147/472)	16.2% (104/640)	10% (1/10)	24.9% (153/613)	24.9% (123/493)	25.5% (176/688)	23.9% (153/640)	6.5% (40/610)	6.4% (34/528)	6% (37/609)	14.6% (115/784)	30.8% (203/659)	31.4% (170/541)	27.2% (48/176)	1% (3/281)	35% (229/653)
*E. coli*	42.3% (11/26)	76.9% (183/238)	28.8% (99/344)	8.4% (29/345)	10% (1/10)	16.4% (58/353)	16.9% (46/272)	20.2% (98/484)	19.3% (79/409)	0.6% (2/356)	0% (0/296)	1.7% (6/345)	8.4% (41/489)	28.2% (111/394)	27.7% (86/311)	29.7% (33/111)	1.2% (2/173)	35.6% (151/424)
*Klebsiella* spp.	57.1% (4/7)	IR	IR	27.9% (46/165)	IR	39% (53/136)	44% (48/109)	43% (61/142)	37.2% (55/148)	13.7% (18/131)	13.7% (16/117)	3.6% (5/137)	18.2% (30/165)	35.6% (48/135)	31.3% (36/115)	17.5% (7/40)	0% (0/75)	30.3% (43/142)
*Serratia* spp.	IR	IR	30% (3/10)	25% (3/12)	NA	30.8% (4/13)	33.3% (4/12)	IR	54.6% (6/11)	30.8% (4/13)	18.2% (2/11)	18.2% (2/11)	38.5% (5/13)	53.8% (7/13)	53.8% (7/13)	NA	100% (1/1)	25% (3/12)
*Proteus* spp.	25% (1/4)	NA	20.8% (10/48)	6.1% (3/49)	NA	22.7% (10/44)	7.7% (3/39)	16% (8/50)	17.9% (10/56)	4.4% (2/45)	0% (0/44)	8% (4/50)	30.4% (17/56)	25% (10/40)	29.3% (12/41)	31.2% (5/16)	IR	45.8% (22/48)
*Pseudomonas* spp.	IR	IR	43.9% (18/41)	29.3% (12/41)	NA	39.5% (15/38)	35.3% (12/34)	IR	IR	32.4% (12/37)	32.4% (11/34)	27% (10/37)	41.7% (15/36)	48.5% (16/33)	50% (17/34)	20% (1/5)	0% (0/24)	IR
*A. baumaniir*	IR	IR	77.8% (7/9)	75% (6/8)	NA	62.5% (5/8)	75% (6/8)	IR	100% (1/1)	28.6% (2/7)	57.1% (4/7)	66.7% (6/9)	71.4% (5/7)	85.7% (6/7)	66.7% (4/6)	100% (1/1)	0% (0/4)	40% (2/5)
*Citrobacter* spp.	IR	IR	25% (1/4)	0% (0/4)	IR	25% (1/4)	33.3% (1/3)	0% (0/3)	0% (0/2)	0% (0/4)	0% (0/3)	0% (0/4)	25% (1/4)	0% (0/4)	0% (0/4)	0% (0/1)	0% (0/1)	25% (1/4)
*M. morganii*	IR	IR	60% (3/5)	20% (1/5)	NA	20% (1/5)	20% (1/5)	NA	20% (1/5)	0% (0/5)	0% (0/4)	0% (0/5)	0% (0/5)	0% (0/4)	20% (1/5)	0% (0/1)	IR	20% (1/5)
*Enterobacter* spp.	IR	IR	16.7% (1/6)	16.7% (1/6)	NA	28.6% (2/7)	14.3% (1/7)	100% (9/9)	12.5% (1/8)	0% (0/7)	0% (0/7)	0% (0/6)	11.1% (1/9)	25% (2/8)	28.6% (2/7)	100% (1/1)	0% (0/3)	12.5% (1/8)
*Providencia*	IR	IR	100% (5/5)	60% (3/5)	NA	80% (4/5)	25% (1/4)	NA	NA	0% (0/5)	20% (1/5)	80% (4/5)	NA	100% (3/3)	100% (5/5)	NA	IR	100% (5/5)

Legend: The percentages represent the resistance. AMC = amoxicillin-clavulanic acid, AMP = ampicillin, PIP = piperacillin, PTZ = piperacillin-tazobactam, TIC = ticarcillin, CFZ = ceftazidime, CFP = cefepime, CFR = cefuroxime, CFX = ceftriaxone, IMI = imipenem, MER = meropenem, AMI = amikacin, GEN = gentamycin, CIP = ciprofloxacin, LEV = levofloxacin, NOR = norfloxacin, COL = colistin, TRS = trimethoprim/sulfamethoxazole, NA = not tested, IR = intrinsic resistance.

**Table 4:** Gram-positive resistance percentages.

	BEN	AMP	OXA	LIN	VAN	GEN	CIP	LEV	NOR	ERY	CLI	TRS
*S. aureus*	90.5% (19/21)	NA	35.3% (6/17)	0% (0/22)	NA	26.3% (5/19)	38.9% (7/18)	NA	50% (1/2)	62.5% (10/16)	60% (12/20)	33.3% (6/18)
*S. agalactiae*	6.4% (3/47)	6.4% (3/47)	NA	0% (0/47)	NA	28.1% (9/32)	NA	44.2% (19/43)	NA	43.7% (14/32)	87.5% (14/16)	NA
*Enterococcus* spp.	NA	16.1% (33/205)	NA	0% (0/202)	1.3% (2/157)	36.7% (73/199)	68% (102/150)	60.3% (94/156)	69.4% (25/36)	NA	NA	NA

Legend: The percentages represent the resistance. BEN = benzylpenicillin, AMP = ampicillin, OXA = oxacillin, LIN = linezolid, VAN = vancomycin, GEN = gentamycin, CIP = ciprofloxacin, LEV = levofloxacin, NOR = norfloxacin, ERY = erythromycin, CLI = clindamycin, TRS = trimethoprim/sulfamethoxazole. NA = not tested.

**Table 5:** Resistance stratified by in and outpatients for gram-negative bacteria.

Bacteria		AMC	AMP	PIP	PTZ	TIC	CFZ	CFP	CFR	CFX	IMI	MER	AMI	GEN	CIP	LEV	NOR	COL	TRS
*E. coli*	IN	NA	**100% (77/77)**	29.3% (94/321)	8.1% (26/321)	12.5% (1/8)	**19.1% (49/256)**	17.4% (44/253)	21.3% (62/291)	**22.6% (65/287)**	0.4% (1/262)	0% (0/277)	1.9% (6/322)	**10.8% (32/297)**	**32.6% (74/227)**	29.6% (69/233)	31.1% (23/74)	0.8% (1/130)	**39.8% (102/256)**
	OUT	42.3% (11/26)	**65.8% (106/161)**	21.7% (5/23)	12.5% (3/24)	0% (0/2)	**9.3% (9/97)**	10.5% (2/19)	18.7% (36/193)	**11.5% (14/122)**	1.1% (1/94)	0% (0/19)	0% (0/23)	**4.7% (9/192)**	**22.2% (37/167)**	21.8% (17/78)	27% (10/37)	2.3% (1/43)	**29.2% (49/168)**
*Klebsiella* spp.	IN	NA	IR	IR	30.4% (42/138)	IR	**43.9% (50/114)**	44% (48/109)	47.5% (56/118)	**42.3% (52/123)**	15.7% (17/108)	13.7% (16/117)	3.7% (5/136)	**23.4% (29/124)**	39.6% (40/101)	**35.4% (34/96)**	23.3% (7/30)	0% (0/71)	33.3% (36/108)
	OUT	57.1% (4/7)	**63.2% (12/19)**	IR	14.8% (4/27)	IR	**13.6% (3/22)**	NA	**20.8% (5/24)**	**12% (3/25)**	4.3% (1/23)	NA	0% (0/1)	**2.4% (1/41)**	23.5% (8/34)	**10.5% (2/19)**	0% (0/10)	0% (0/4)	20.6% (7/34)
*Proteus* spp.	IN	NA	NA	17.8% (8/45)	4.3% (2/46)	NA	25% (9/36)	8.1% (3/37)	13.2% (5/38)	18.2% (8/44)	5.3% (2/38)	0% (0/41)	8.5% (4/47)	**38.1% (16/42)**	27.6% (8/29)	29.4% (10/34)	33.3% (4/12)	IR	45.9% (17/37)
	OUT	25% (1/4)	NA	66.7% (2/3)	33.3% (1/3)	NA	12.5% (1/8)	0% (0/2)	25% (3/12)	16.7% (2/12)	0% (0/7)	0% (0/3)	0% (0/3)	**7.1% (1/14)**	18.2% (2/11)	28.6% (2/7)	25% (1/4)	IR	45.5% (5/11)

Legend: The percentages represent the resistance. AMC = amoxicillin-clavulanic acid, AMP = ampicillin, PIP = piperacillin, PTZ = piperacillin-tazobactam, TIC = ticarcillin, CFZ = ceftazidime, CFP = cefepime, CFR = cefuroxime, CFX = ceftriaxone, IMI = imipenem, MER = meropenem, AMI = amikacin, GEN = gentamycin, CIP = ciprofloxacin, LEV = levofloxacin, NOR = norfloxacin, COL = colistin, TRS = trimethoprim/sulfamethoxazole, IR = intrinsic resistance, NA = not evaluated, IN = inpatients, OUT = outpatients. Bolded values are statistically different—Chi-square test.

**Table 6:** Resistance stratified by in and outpatients for Gram-positive bacteria.

		AMP	VAN	GEN	CIP	LEV	NOR	ERY	CLI
*Enterococcus* spp.	IN	17.8% (31/174)	1.5% (2/135)	**41.7% (70/168)**	**72.1% (93/129)**	**65.9% (87/132)**	**76.7% (23/30)**	80% (4/5)	5.9% (1/17)
	OUT	6.5% (2/31)	0% (0/22)	**9.7% (3/31)**	**42.9% (9/21)**	**29.2% (7/24)**	**33.3% 9/26)**	NA	0% (0/2)

Legend: The percentages represent the resistance. NA = not evaluated, IN = inpatients, OUT = outpatients, bolded values are statistically different—chi-square test.

**Table 7:** Classification of the most common bacterial uropathogens into resistance phenotypes.

Bacterial Species	ESBL	CRO	R-SXT	R-AG	R-FQ	BL	MDR	XDR
*Escherichia coli*	16.9% (46/272)	0.5% (2/374)	35.6% (151/424)	8.1% (42/520)	28.3% (147/519)	36.3% (193/532)	22.1% (118/534)	1.5% (8/534)
*Klebsiella* spp.	44% (48/109)	14.1% (20/142)	30.3% (43/142)	17% (31/182)	30.9% (55/178)	23.1% (43/186)	32.2% 60/186	4.8% 9/186
*Proteus* spp.	7.7% (3/39)	4.2% (2/48)	45.8% (22/48)	27.4% (17/62)	27% (17/63)	61.7% (37/60)	12.7% (8/63)	4.7% (3/63)
*Pseudomonas* spp.	35.3% (12/34)	26.7% (12/37)	IR	40% (16/40)	46.3% (19/41)	100% (41/41)	36.5% (15/41)	26.8% (11/41)
*Serratia* spp.	33.3% (4/12)	30.8% (4/13)	25% (3/12)	38.5% (5/13)	53.8% (7/13)	23.1% (3/13)	23.1% (3/13)	15.3% (2/13)
*Enterobacter* spp.	14.3% (1/7)	0% (0/7)	12.5% (1/8)	11.1% (1/9)	33.3% (3/9)	NA	11.1% (1/9)	11.1% (1/9)
*Acinetobacter baumannii*	75% (6/8)	57.1% (4/7)	40% (2/5)	66.7% (6/9)	77.8% (7/9)	100% (9/9)	66.6% (6/9)	44.4% (4/9)
*Morganella morganii*	20% (1/5)	0%	20% (1/5)	0%	16.7% ()1/6	IR	16.7% (1/6)	0%
*S. aureus*	NA	NA	33.3% (6/18)	26.3% (5/19)	40% (8/20)	90.5% (19/21)	63.6% (14/22)	0%
*S. agalactiae*	NA	NA	IR	28.1% (9/32)	44.2% (19/43)	NA	0%	0%
*Enterococcus* spp.	NA	NA	IR	36.7% (73/199)	68.7% (134/195)	12.2% (24/196)	11.7% 24/205	0%

Legend: ESBL = Extended-spectrum beta-lactamases producing Gram-negative bacilli, CRO = carbapenem-resistant organisms, R-SXT = resistance to sulfonamides, R-AG = resistance to aminoglycosides, R-FQ = resistance to fluoroquinolones, MDR = multidrug-resistant bacteria, XDR = extensively drug-resistant bacteria, IR = intrinsic resistance, NA = not applicable.

## Data Availability

The original contributions presented in this study are included in the article/Supplementary Material, and further inquiries can be directed to the corresponding author at the e-mail address chisavu.lazar@umft.ro. The raw data supporting the conclusions of this article will be made available by the authors upon request. All of the data are presented in the current form of the manuscript.

## Supplementary Materials

The following supporting information can be downloaded at: https://www.mdpi.com/article/10.3390/diseases13030074/s1, Table S1: Panel of antibiotics tested on Vitek cardstitle; Table S2: Panel of antibiotics tested by disk diffusion.

## References

[1] Antimicrobial Resistance Collaborators (2022). Global burden of bacterial antimicrobial resistance in 2019: A systematic analysis. Lancet.

[2] Cassini A., Högberg L.D., Plachouras D., Quattrocchi A., Hoxha A., Simonsen G.S., Colomb-Cotinat M., Kretzschmar M.E., Devleesschauwer B., Cecchini M. (2019). Attributable deaths and disability-adjusted life-years caused by infections with antibiotic resistant bacteria in the EU and the European Economic Area in 2015: A population-level modelling analysis. Lancet Infect. Dis..

[3] Lim C., Takahashi E., Hongsuwan M., Wuthiekanun V., Thamlikitkul V., Hinjoy S., Day N.P., Peacock S.J., Limmathurotsakul D., Hospital A. (2016). Epidemiology and burden of multidrug-resistant bacterial infection in a developing country. eLife.

[4] Vos T., Lim S.S., Abbafati C., Abbas K.M., Abbasi M., Abbasifard M., Abbasi-Kangevari M., Abbastabar H., Abd-Allah F., Abdelalimet A. (2020). Global burden of 369 diseases and injuries in 204 countries and territories, 1990–2019: A systematic analysis for the Global Burden of Disease Study 2019. Lancet.

[5] de Kraker M.E.A., Stewardson A.J., Harbarth S. (2016). Will 10 million people die a year due to antimicrobial resistance by 2050?. PLoS Med..

[6] Bassetti S., Tschudin-Sutter S., Egli A., Osthoff M. (2022). Optimizing antibiotic therapies to reduce the risk of bacterial resistance. Eur. J. Intern. Med..

[7] Petca R.-C., Mareș C., Petca A., Negoiță S., Popescu R.-I., Boț M., Barabás E., Chibelean C.B. (2020). Spectrum and Antibiotic Resistance of Uropathogens in Romanian Females. Antibiotics.

[8] Petca R.-C., Negoiță S., Mareș C., Petca A., Popescu R.-I., Chibelean C.B. (2021). Heterogeneity of Antibiotics Multidrug-Resistance Profile of Uropathogens in Romanian Population. Antibiotics.

[9] Chibelean C.B., Petca R.-C., Mareș C., Popescu R.-I., Enikő B., Mehedințu C., Petca A. (2020). A Clinical Perspective on the Antimicrobial Resistance Spectrum of Uropathogens in a Romanian Male Population. Microorganisms.

[10] Arbune M., Gurau G., Niculet E., Iancu A.V., Lupasteanu G., Fotea S., Vasile M.C., Tatu A.L. (2021). Prevalence of antibiotic resistance of ESKAPE pathogens over five years in an infectious diseases hospital from South-East of Romania. Infect. Drug Resist..

[11] Farkas A., Tarco E., Butiuc-Keul A. (2019). Antibiotic resistance profiling of pathogenic Enterobacteriaceae from Cluj-Napoca, Romania. Germs.

[12] Mareș C., Petca R.-C., Popescu R.-I., Petca A., Mulțescu R., Bulai C.A., Ene C.V., Geavlete P.A., Geavlete B.F., Jinga V. (2024). Update on Urinary Tract Infection Antibiotic Resistance—A Retrospective Study in Females in Conjunction with Clinical Data. Life.

[13] Chiţă T., Timar B., Muntean D., Bădiţoiu L., Horhat F., Hogea E., Moldovan R., Timar R., Licker M. (2016). Urinary tract infections in Romanian patients with diabetes: Prevalence, etiology, and risk factors. Ther. Clin. Risk Manag..

[14] Sorescu T., Licker M., Timar R., Musuroi C., Muntean D., Voinescu A., Vulcanescu D.D., Cosnita A., Musuroi S.-I., Timar B. (2024). Characteristics of Urinary Tract Infections in Patients with Diabetes from Timișoara, Romania: Prevalence, Etiology, and Antimicrobial Resistance of Uropathogens. Medicina.

[15] Ny S., Edquist P., Dumpis U., Gröndahl-Yli-Hannuksela K., Hermes J., Kling A.M., Klingeberg A., Kozlov R., Källman O., Lis D.O. (2019). NoDARS UTIStudy Group. Antimicrobial resistance of Escherichia coli isolates from outpatient urinary tract infections in women in six European countries including Russia. J. Glob. Antimicrob. Resist..

[16] Isac R., Doros G., Stolojanu C.-A., Steflea R.M., Stroescu R.F., Olariu I.-C., Micsescu-Olah A.-M., Gafencu M. (2024). General Characteristics and Current State of Antibiotic Resistance in Pediatric Urinary Tract Infection—A Single Center Experience. Antibiotics.

[17] Clinical and Laboratory Standards Institute (CLSI) (2020). Performance Standards for Antimicrobial Susceptibility Testing.

[18] Clinical and Laboratory Standards Institute (CLSI) (2021). Performance Standards for Antimicrobial Susceptibility Testing.

[19] Magiorakos A.P., Srinivasan A., Carey R.B., Carmeli Y., Falagas M.E., Giske C.G., Harbarth S., Hindler J.F., Kahlmeter G., Olsson-Liljequist B. (2012). Multidrug-resistant, extensively drug-resistant and pandrug-resistant bacteria: An international expert proposal for interim standard definitions for acquired resistance. Clin. Microbiol. Infect..

[20] Litwin M.S., Saigal C.S., Yano E.M., Avila C., Geschwind S.A., Hanleym J.M., Joycem G.F., Madison R., Pace J., Polich S.M. (2005). Urologic diseases in America project: Analytical methods and principal findings. J. Urol..

[21] Royal College of General Practitioners, Office of Population Censuses and Surveys, Department of Health (1995). Morbidity Statistics from General Practice: Fourth National Study 1991–1992.

[22] Cuiban E., Radulescu D., David C., Turcu F.L., Bogeanu C., Feier L.F., Onofrei S.D., Vacaroiu I.A. (2023). Wcn23-0468 urinary tract infections in chronic kidney disease patients—A Romanian centre experience. Kidney Int. Rep..

[23] Khameneh B., Diab R., Ghazvini K., Bazzaz B.S.F. (2016). Breakthroughs in bacterial resistance mechanisms and the potential ways to combat them. Microb. Pathog..

[24] Khan A., Saraf V.S., Siddiqui F., Batool T., Noreen Z., Javed S., Ahmad A., Alonazi W.B., Ibrahim M., Pucciarelli S. (2024). Multidrug resistance among uropathogenic clonal group A E. Coli isolates from Pakistani women with uncomplicated urinary tract infections. BMC Microbiol..

[25] Amin A., Noureen R., Iftikhar A., Hussain A., Alonazi W.B., Raza H.M.Z., Ferheen I., Ibrahim M. (2024). Uropathogenic bacteria and deductive genomics towards antimicrobial resistance, virulence, and potential drug targets. Int. Microbiol..

[26] Reardon S. (2015). Dramatic rise seen in antibiotic use. Nature.

[27] https://www.thebusinessresearchcompany.com/report/multidrug-resistant-bacteria-global-market-report.

[28] Firth A., Prathapan P. (2021). Broad-spectrum therapeutics: A new antimicrobial class. Curr. Res. Pharmacol. Drug Discov..

[29] Magill S.S., Edwards J.R., Bamberg W., Beldavs Z.G., Dumyati G., Kainer M.A., Lynfield R., Maloney M., McAllister-Hollod L., Nadle J. (2014). Emerging Infections Program Healthcare-Associated Infections and Antimicrobial Use Prevalence Survey Team. Multistate point-prevalence survey of health care-associated infections. N. Engl. J. Med..

