# Supplementary material for: In and Outpatients Bacteria Antibiotic Resistances in Positive Urine Cultures from a Tertiary Care Hospital in the Western Part of Romania—A Cross-Sectional Study
Source: Diseases. 2025 Mar 1;13(3):74. doi: 10.3390/diseases13030074 (PMC11941544; doi:10.3390/diseases13030074)
Supplement: Supplementary file 1 [file diseases-13-00074-s001.zip › diseases-3455967-supplementary.pdf]

Suppl. Table S1: Panel of antibiotics tested on Vitek cards

Card Type	Antibiotics	Pathogens tested
AST-N222	Piperacillin	GNB –Enterobacterales, non-fermenters
	Ticarcillin/Clavulanic Acid	GNB –Enterobacterales, non-fermenters
	Piperacillin/Tazobactam	GNB –Enterobacterales, non-fermenters
	Ceftazidime	GNB –Enterobacterales, non-fermenters
	Cefepime	GNB –Enterobacterales, non-fermenters
	Aztreonam	GNB –Enterobacterales, non-fermenters
	Imipenem	GNB –Enterobacterales, non-fermenters
	Meropenem	GNB –Enterobacterales, non-fermenters
	Amikacin	GNB –Enterobacterales, non-fermenters
	Gentamicin	GNB –Enterobacterales, non-fermenters
	Tobramycin	GNB –Enterobacterales, non-fermenters
	Ciprofloxacin	GNB –Enterobacterales, non-fermenters
	Colistin	GNB –Enterobacterales, non-fermenters
	Trimethoprim/Sulfamethoxazole	GNB –Enterobacterales, Acinetobacter
AST-GP67	Oxacillin	*Staphylococcus aureus*
	Benzylpenicillin	*Staphylococcus aureus*
	Ampicillin	*Enterococcus*
	Linezolid	*Staphylococcus aureus , Enterococcus*
	Vancomycin	*Staphylococcus aureus, Enterococcus*
	Tetracycline	*Staphylococcus aureus*
	Tigecycline	*Staphylococcus aureus, Enterococcus*
	Gentamicin	*Staphylococcus aureus*
	Gentamicin High Level	*Enterococcus*
	Streptomycin High Level	*Enterococcus*
	Ciprofloxacin	*Staphylococcus aureus ,Enterococcus*
	Levofloxacin	*Staphylococcus aureus , Enterococcus*
	Moxifloxacin	*Staphylococcus aureus*

	Quinupristin/Dalfopristin	*Staphylococcus aureus , Enterococcus*
	Erythromycin	*Staphylococcus aureus*
	Clindamycin	*Staphylococcus aureus*
	Trimethoprim/Sulfamethoxazole	*Staphylococcus aureus*
	Rifampicin	*Staphylococcus aureus*

Suppl. Table S2: Panel of antibiotics tested by disk diffusion

Panel of antibiotics	Antibiotics	Pathogen tested
Gram negative panel of antibiotics	Piperacillin	GNB –Enterobacterales, non-fermenters
	Ampicillin	GNB –Enterobacterales
	Amoxicillin+clavulanic acid,	GNB –Enterobacterales
	Piperacillin/Tazobactam	GNB –Enterobacterales, non-fermenters
	Cefuroxime	GNB –Enterobacterales
	Ceftazidime	GNB –Enterobacterales, non-fermenters
	Ceftriaxone	GNB –Enterobacterales
	Cefepime	GNB –Enterobacterales, non-fermenters
	Ciprofloxacin	GNB –Enterobacterales, non-fermenters
	Levofloxacin	GNB –Enterobacterales, non-fermenters
	norfloxacin,	GNB –Enterobacterales, non-fermenters
	Nitrofurantoin	GNB –Enterobacterales
	Imipenem	GNB –Enterobacterales, non-fermenters
	Meropenem	GNB –Enterobacterales, non-fermenters
	Amikacin	GNB –Enterobacterales, non-fermenters
	Gentamicin	GNB –Enterobacterales, non-fermenters
	Trimethoprim/Sulfamethoxazole	GNB –Enterobacterales*, Acinetobacter*
Gram pozitive panel of antibiotics	Cefoxitine	*Staphylococcus aureus*
	Benzylpenicillin	*Staphylococcus aureus, Corynebacterium*

	Ampicillin	*Enterococcus*
	Linezolid	*Staphylococcus aureus , Enterococcus, Corynebacterium*
	Vancomycin	*Enterococcus, Corynebacterium*
	Gentamicin	*Staphylococcus aureus*
	Gentamicin High Level	*Enterococcus*
	Ciprofloxacin or Levofloxacin or Norfloxacin	*Staphylococcus aureus ,Enterococcus, Corynebacterium*
	Erythromycin	*Staphylococcus aureus*
	Clindamycin	*Staphylococcus aureus, Corynebacterium*
	Trimethoprim/Sulfamethoxazole	*Staphylococcus aureus*

Supplementary material and methods:

Sample collection

Urine is collected in a sterile container, cup with lid. It is recommended to collect the first-morning urine or at least 3 hours after previous urination, after proper toilet of external genital organs. The first stream of urine is allowed to flow in the toilet, then 10-20 ml of urine from the middle stream is collected in the sterile container, without touching the inside of the bottle. If a patient presented a urine catheter, the catheter was changed with a new one, and afterwards the sample was collected. After collection, the container is carefully closed to ensure tightness; the bar code label is stuck on the container. The sample have to be transported to the laboratory within 2 hours. The samples collected from in and outpatients are transported to the laboratory within 2 hours by the ward medical staff, in closed, isothermal bags to ensure sample integrity and the safety of the transporter. Urine collection is done outside an antibiotic treatment, or at least 3 days after the end of the treatment; when the patient is under antibiotic treatment, this must be specified, as well as the antibiotic administered.

Bacterial Identification and Antibiotic Testing

Samples were then transported and processed within 2 hours (or if processing was delayed up to 24 hours, the urine was refrigerated). In the Microbiology laboratory Gram smear microscopic examination of uncentrifuged urine was performed. Then, calibrated loop method was used for insemination. The end of a sterile 10µL calibrated loop was dipped in urine and 2 plates were inoculated (Mac Conckey agar medium and chromogenic agar) followed by homogeneous urine dispersion. Incubation was performed 24-48h at 35-37°C. Urines with more than 105 colony-forming units/mL (CFU/mL) were considered positive and evaluated. For interpretation, a colony was considered equal to 100 CFU/mL.. The identification was performed on VITEK® 2 Compact (BioMerieux, Marcy l'Etoile, France) and matrix-assisted laser ionization/desorption mass spectrometry on-flight mass spectrometry (MALDI Biotyper, Bruker, Germany) systems.

For urine samples collected from patients hospitalized in wards where antimicrobial resistance (AMR) is usually reaching increased rates (such as intensive care, burn and surgical wards), Antimicrobial susceptibility tests AST VITEK 2 -N222 and VITEK 2 AST GP 67 cards, (BioMérieux, Marcy l'Etoile, France) were used by determining the minimum inhibitory concentration (MIC). In contrast, uropathogens identified in patients hospitalized in the hospital medical wards or outpatients, where lower levels of AMR are recorded, were tested by the Kirby-Bauer disk diffusion method. In this case, antibiotics from Thermo Fischer Scientific were used.
